# Comparing supervised and unsupervised approaches to emotion categorization in the human brain, body, and subjective experience

**DOI:** 10.1038/s41598-020-77117-8

**Published:** 2020-11-20

**Authors:** Bahar Azari, Christiana Westlin, Ajay B. Satpute, J. Benjamin Hutchinson, Philip A. Kragel, Katie Hoemann, Zulqarnain Khan, Jolie B. Wormwood, Karen S. Quigley, Deniz Erdogmus, Jennifer Dy, Dana H. Brooks, Lisa Feldman Barrett

**Affiliations:** 1 Department of Electrical & Computer Engineering, College of Engineering, Northeastern University, Boston, MA USA; 2 Department of Psychology, College of Science, Northeastern University, Boston, MA USA; 3 Department of Psychology, University of Oregon, Eugene, OR USA; 4 Institute of Cognitive Science, University of Colorado Boulder, Boulder, CO USA; 5 Department of Psychology, University of New Hampshire, Durham, NH USA; 6 Edith Nourse Rogers Veterans Hospital, Bedford, MA USA; 7 Department of Psychiatry, Massachusetts General Hospital, Boston, MA USA; 8 Athinoula A. Martinos Center for Biomedical Imaging, Massachusetts General Hospital, Boston, MA USA

**Keywords:** Human behaviour, Neuroscience, Psychology, Scientific data

## Abstract

Machine learning methods provide powerful tools to map physical measurements to scientific categories. But are such methods suitable for discovering the ground truth about psychological categories? We use the science of emotion as a test case to explore this question. In studies of emotion, researchers use supervised classifiers, guided by emotion labels, to attempt to discover biomarkers in the brain or body for the corresponding emotion categories. This practice relies on the assumption that the labels refer to objective categories that can be discovered. Here, we critically examine this approach across three distinct datasets collected during emotional episodes—measuring the human brain, body, and subjective experience—and compare supervised classification solutions with those from unsupervised clustering in which no labels are assigned to the data. We conclude with a set of recommendations to guide researchers towards meaningful, data-driven discoveries in the science of emotion and beyond.

## Introduction

Psychology became a science in the mid-19th century when scholars first used the research methods of physiology and neurology to search for the physical basis of mental categories that were inherited from ancient Western mental philosophy—categories that describe thinking, feeling, perceiving and acting. Scientific leaders of the day (e.g.^1,2^) warned that these common-sense categories, which philosophers call “folk psychology”^3–7^, would not map cleanly to physical measurements. Nonetheless, scientists have persisted for more than a century in their attempts to map measurements of the brain, body, and behavior to common-sense mental categories for cognitions, emotions, perceptions and so on. Many empirical efforts within psychological science rely on the assumption that Western folk category labels constitute the biological and psychological ‘ground truth’ of the human mind across cultures (e.g., mapping categories such as attention, emotion, or cognition onto functional brain networks^8^), while other efforts are more neutral in their assumptions, inferring only that the category characterizes a participant’s behavior (e.g., rating of experience conditioned on experimenter-provided folk labels^9^). Recently, however, a growing number of scientists have questioned the utility of folk category labels for organizing a science of the mind and behavior, noting the persistent difficulty of cleanly mapping these categories onto measurements of brain activity, physiological changes in the body, and behavior^10–18^.

In this paper, we re-examine one aspect of the hypothesis that a natural description of the human mind—i.e., the biological organization of behavior and human experience—may require stepping back from the assumption that folk categories describe the ground truth. We do this in the context of machine learning to examine how unsupervised and supervised machine learning approaches can be used to build interpretative models from psychology measurements, across three quite different experimental settings. Unsupervised machine learning approaches can discover meaningful structure in data without assigning labels, providing a potentially valuable tool for scientific discovery in mapping biology to psychology. Supervised learning, by contrast, looks for structure in data that matches assigned labels. By comparing the results of supervised and unsupervised machine learning analyses, we can assess the extent to which psychological categories can reasonably be considered the ground truth for what exists in some objective way. Our goal is to demonstrate the importance of critically examining the labels being used, not to explain the discrepancies between supervised and unsupervised solutions or to provide evidence for a particular hypothesis. This is a proof-of-concept paper, rather than a strong inference paper, with the goal of encouraging researchers to start approaching psychology in a somewhat different way, regardless of theoretical stance. We are not suggesting that theory is unimportant in psychological science. We are instead hypothesizing that focusing empirical efforts on folk categories and building theory around those may not be the best (or only) approach. We use emotion categories as our example because they are complex phenomena that are hypothesized to be organized as a taxonomy, and there have been numerous debates about the “ground-truth” categories. As a consequence, the domain of emotion is a clear test case in which labels that enforce strict category boundaries are traditionally imposed on the data in supervised classification methods. Whether these boundaries exist and can be discovered using unsupervised approaches is highly debated (for a discussion, see^19,20^). We selected datasets that were representative of current published research being conducted in the science of emotion, covered a range of induction techniques that have been shown to be effective^21^ and common measurement modalities (self-report, psychophysiology, and fMRI), and were available to us in sufficient quantity such that variability could be adequately assessed. We present findings from supervised and unsupervised analyses of three existing datasets and show that, across all three datasets, supervised and unsupervised methods do not produce concordant results. We conclude the paper with a set of recommendations and future directions for machine learning investigations to guide researchers towards using unsupervised methods to discover biologically meaningful and reproducible discoveries about the nature of emotion and the human mind more generally.

### Emotion categories: the example

In the last decade, a number of researchers have applied supervised machine learning techniques to various types of data in an attempt to build classifiers that can identify ‘biomarkers,’ ‘signatures,’ or ‘fingerprints’ for pre-defined mental categories (for a review, see^22^). The science of emotion provides a particularly useful example of the problems encountered when using this approach to search for the physical basis of the human mind. Scientists begin with emotion categories for anger, sadness, fear, and so on, and then select stimuli, such as scenarios, movie clips, or music, that they expect will evoke the most frequent or common instances of each category (see Table 1 for details of previous studies). These studies typically select stimuli that optimally differentiate emotion categories, and participants are exposed to these stimuli while the experimenters take measurements of their brains, bodies, and/or behavior. Scientists then aim to find evidence in the observed data for the relevant emotion categories by using supervised machine learning methods. Classifiers that are sensitive and specific to a distinct emotion category across participants and contexts^22^ are then taken as evidence that the patterns or class representatives that those classifiers determine are the biomarkers of the categories. Guided by this logic, several recent studies have reported that they have identified unique patterns of brain activity, autonomic nervous system (ANS) changes, or subjective experience that discriminate emotion categories such as fear, anger, sadness, happiness, etc.^9,23–32^. To the extent that these studies have identified markers that are truly representative of different emotion categories, and generalize across populations and contexts, these studies may be taken by researchers as evidence that emotion categories have a reliable biological and mental basis.

However, a natural description of the human mind (i.e., the biological organization of behavior and human experience) may require stepping back from the assumption that folk categories describe the ground truth about emotions (or indeed any psychological domain). There are at least two reasons why the science of emotion is a particularly good test case to explore this question. First, the assumption that a single solution will serve as the best classifier across situations, stimuli, samples, and so on, is inherent in the language and modeling approaches used (e.g., using a single label per instance rather than multiple labels, often using deterministic rather than probabilistic models, etc; see Table 1) A close look at the published findings so far reveals that the patterns reported to be associated with a given emotion category, such as ‘fear,’ have not been consistent across studies. For example, individual studies report patterns of ANS activity that distinguish one emotion category from another, but the actual patterns vary across studies for a given emotion category, even when the studies in question use the same methods and stimuli and sample from the same population of participants (e.g.,^25,31^). Similar cross-study, within-category variation is observed for multivoxel pattern analysis (MVPA) of blood oxygen level dependent (BOLD) signals across the brain (e.g., compare^26,29,32^; for a discussion, see^16^). There are several possible reasons why this has been the case. Methodological considerations might explain variation in these classification-based results^33,34^. For example, in brain imaging studies, small sample sizes, different affect induction methods, issues with the alignment of brains across participants, variable preprocessing workflows, and the use of variable classification algorithms might all contribute to the instability of solutions across studies (see Table 1). It is also possible that current functional brain imaging measures are insufficiently sensitive or comprehensive to identify the biomarker for a given emotion category. There may also be more than one biomarker for each category, or perhaps a single biomarker exists but multiple models capture this ground truth in different ways. But it may also be that the relationship between the presumed emotion categories, used to generate the labels for the analysis and the physiological response measured in the body or brain, are simply more complex than can be fit with a consistent relationship between the two. A purely methodological explanation may be insufficient for explaining the variation in classification-based results across studies, however, given that methodological advances for over a century have not substantially reduced the considerable variation that is observed within an emotion category across different measurement methods yielding different types of data (e.g., self-report, physiology, and brain imaging). Instead, the within-category variation that has been observed for decades (see^20,35^) is consistent with the variation observed in classification-based results, and therefore supports the hypothesis that there is an opportunity to discover something meaningful about the nature of emotion. Specifically, it suggests at least two avenues of inquiry; one would be to systematically test for one or more models across multiple datasets and modalities given a categorization. Another is to look more carefully at the relationship between the structure implied by the labels and the intrinsic structure of the acquired data across different modalities; it is an approach towards the latter inquiry that we describe here.

**Table 1:** Summary of past MVPA studies of BOLD data.

Study	N	# Emot. categories	# Stimuli per category (unique)	Induction method	Relevant preprocessing	Feature selection	Classification algorithm
Kassam et al.^24^	10	9	2	Participant-generated scenario immersion	No spatial smoothing	Voxels with the most stable activation profile	Gaussian Naive Bayes
					Z-scoring		
					Anatomical normalization		
Kragel and LaBar^25^	32	7	4	Movies Music	No spatial smoothing	All grey matter voxels	Partial least squares discriminant analysis
					Mean centering		
					Anatomical normalization		
Saarimäki et al.^29^	48	5 (study 1) 6 (study 2)	10 (study 1) 6 (study 2)	Participant-generated scenario immersion Movies	No spatial smoothing	Voxels most sensitive to manipulation using ANOVA	Linear neural network with no hidden layers
					Z-scoring		
					Anatomical normalization		
Saarimäki et al.^30^	25	15	4	Narrative-guided scenario immersion	No spatial smoothing	Voxels most sensitive to manipulation using ANOVA	Linear neural network with no hidden layers
					Z-scoring		
					Anatomical normalization		
Wager et al.^32^	2159	5	Variable	Variable	Meta-analysis (peak based)	Whole brain	Bayesian spatial point process model
					Binarized data		
					Anatomical normalization		

These studies all claim to identify unique patterns of brain activity for specific emotion categories, yet these patterns are inconsistent across studies. Other existing MVPA studies of affect (e.g.,^38^), and conceptual knowledge (e.g.,^39^) are less relevant and so are not listed here.

A second reason to question whether biology and behavior are best accounted for by a single set of emotion categories, each with a single biomarker, comes from evidence of considerable within-category variability and cross-category similarity in measurements of neural activity, ANS activity, and behavior. For example, in the domain of peripheral physiology, measurements of heart period or respiration are tied to the metabolic demands that support current or predicted actions in a given situation (e.g., cardiac output goes up when a person is about to run, but not when a person freezes and is vigilant for more information to resolve uncertainty or ambiguity^40,41^). People vary in their physical actions across instances of the same emotion category that occur in different situations (e.g., when experiencing fear, a person may run away, freeze, or attack); as a consequence, instances of fear will vary in their physiological features^42^. Similar within-category variation and between-category similarities have been observed in expressive facial movements^43,44^, and in neural correlates, including magnitude of the BOLD response^36,45,46^, functional connectivity^47,48^, and single neuron recordings^49^. Instances of an emotion category also vary in their affective features (e.g., some instances of fear can feel pleasant and some instances of happiness can feel unpleasant^46^. This magnitude of variation, which has been replicated numerous times in the study of emotion over the past century^50^ strengthens the possibility that imposing a single label on data may restrict or even obscure the discovery of meaningful, alternative categories in the emotion domain.

With these observations in mind, we ask whether the use of predetermined labels in building supervised classifiers might be contributing to the apparent contradiction between above-chance classification and variation across studies. Specifically, we examined whether unsupervised clustering provides a useful alternative to supervised clustering in the discovery of meaningful categories when studying the domain of emotion. Our unsupervised clustering methods consistently treat the number of clusters as a statistical parameter to be learned which, in principle, allows researchers to discover a more flexible and variable category structure should it exist, while also discovering similarities across people, should those exist. We present findings from supervised and unsupervised analyses applied to three rather diverse datasets on emotion: (1) an archival dataset during which 16 participants immersed themselves in auditory scenarios for instances labeled as inducing happiness, sadness, and fear during functional magnetic resonance imaging (fMRI)^51^, (2) an ambulatory peripheral physiology study during which peripheral physiological signals were recorded from 46 participants throughout daily life; physiological changes (specifically, a change in interbeat interval, the time between heartbeats) triggered self-report measurement episodes in which participants freely labeled their emotional experiences^52^, and (3) a publicly available self-report dataset in which 853 participants viewed a subset of 2185 movie clips and reported on their experiences with categorical (yes/no) ratings of 34 emotion words or Likert ratings of 14 affective dimensions^9^. We subjected each dataset to a supervised classification approach with emotion category labels, as well as an unsupervised, data-driven approach which included statistically validated methods to determine the number of clusters that are objectively supported by the data. Because it is difficult to ask the same question across three different datasets that have been collected in different ways, with different constraints, we analyzed each dataset with the supervised and unsupervised approaches that were most appropriate for that particular data, while trying to the extent feasible to keep the tenets and features of the analyses consistent.

## Results

### Example 1: blood oxygen level dependent (BOLD) data from Wilson-Mendenhall et al.^51^

Our first set of analyses involved an archival dataset^51^ from a study during which sixteen participants underwent fMRI scanning while immersing themselves in auditory scenarios labeled by experimenters according to their intention to induce experiences of happiness, sadness, or fear. Participants heard a total of 60 scenarios associated with each emotion category, for a total of 180 trials across 6 runs of scanning. We analyzed whole-brain blood oxygen level dependent (BOLD) signals from 9 s windows during which participants listened to, and immersed themselves in, a single auditory scenario. Additional participant demographic and task details are reported in the “Methods” section. Evidence suggests that participants were simulating highly embodied emotional experiences when immersing themselves in the auditory scenarios. Wilson-Mendenhall et al.^53^ analyzed the same dataset used in Example 1 and observed increased activity in primary motor cortex and premotor cortex and in primary somatosensory cortex while participants were lying still in the scanner, and increased activity in primary visual cortex while participants’ eyes were closed. The researchers also observed increased activity in brain areas that are typically considered to be important for emotion, such as the ventromedial prefrontal cortex and the anterior insula, as well as primary interoceptive cortex, the thalamus, hypothalamus, and subcortical nuclei that regulate the autonomic nervous system, immune system, and metabolic systems.

**Figure 1:**
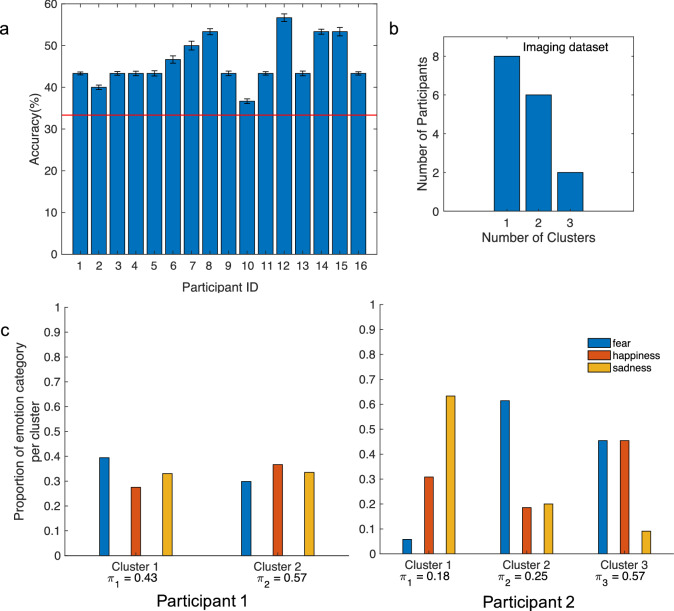
[BOLD Data] Results from supervised and unsupervised analyses of BOLD data from the 9 s scenario immersion. (**a**) Supervised clustering: Mean ± SEM classification accuracy from within-subject CNN supervised classification. The red line represents chance level accuracy (33.3%). (**b**,**c**) Unsupervised clustering: (**b**) Histogram of number of participants fit by 1, 2, or 3 GMM clusters. Specifically, eight participants had one discovered cluster, six had two, and two had three. (**c**) Correspondence between discovered clusters and emotion category labels for two example participants (corresponding to Participant IDs 1 and 2 in part **a**) with two (left) and three (right) discovered clusters respectively. Bars represent the proportion of the trials from each emotion category found within each cluster. Blue bars represent trials labeled as fear, orange bars happiness, and yellow sadness. The total proportion of categories in each cluster sums to 1. The mixing proportion $\pi _k$ reported below each cluster is the probability that an observation comes from that cluster, which is representative of the size of the cluster.

To study whether a supervised classifier could recover the experimenter labels, we first conducted supervised classification of the BOLD data to examine classifier performance when using the folk emotion labels. Specifically, we used a 3D Convolutional Neural Network^54^ with sixfold cross validation, iterating across all combinations of training on each participant’s data from five runs and testing on a left-out run (see Supplementary Materials for classifier details). Mean within-participant accuracy was calculated by averaging the accuracy across all cross-validation folds. Chance level was defined as one divided by the number of categories, or 1/3 (33.3%), and the mean accuracy was compared to chance performance using a one-sample t-test. The classifier achieved a statistically significant above-chance mean within-participant accuracy of 46.06% ($t(15) = 9.07$, $p<0.01$; Fig. 1a).

We next used an unsupervised clustering method—a Gaussian Mixture Model (GMM^55^)—that allowed us to statistically determine the number of clusters which best described the data for each participant. GMMs have been used to successfully cluster BOLD data in several domains of research (e.g.,^56–58^). To validate the sensitivity of the GMM to detect true categories in the data, we first tested our model using synthetic data generated according to a generative process described in^59^. The synthetic data were designed to imitate BOLD data with clear, discoverable categories (which were classified above-chance when using a supervised approach; Supplementary Fig. 5a). We included this validation step to ensure that the GMM would be able to successfully cluster the data if the relevant signal was present. Specifically, for each emotion category, we considered several randomly chosen regions in the brain with varying BOLD amplitude during each trial. Each of these regions was assigned a single radial basis function to generate a BOLD amplitude whose spatial center and width was chosen uniformly within limits of a standard human brain. The synthesized brain image for each trial was a weighted combination of these basis functions for the specific emotion category active during that trial. Zero-mean Gaussian noise was added at the last step to account for measurement noise. We then tested the sensitivity of our GMM model on the synthetic BOLD data using a concatenation of Principal Components Analysis (PCA^60^) to reduce the dimensionality of the data followed by the Gaussian Mixture Model (GMM) to discover clusters in the lower-dimensional data (using Bayesian Information Criteria (BIC^61^) to jointly estimate the number of PCA components and GMM clusters that best described the data). We found that the GMM was able to discover clusters in the synthetic data (Supplementary Fig. 5b). To measure our model’s ability to detect clusters in the synthetic data, we labeled the discovered clusters by the most prevalent label of the data within that cluster and then compared the proportion of samples within a given cluster whose category labels corresponded to the assigned cluster label. Even at extremely low (indeed, unlikely, given values reported in the literature ranging from 0.35 to 203.6^37^) signal-to-noise ratio (SNR) values, the accuracy of the model was higher than chance (accuracy was 40% at an unlikely SNR of $10^{-2}$, compared to chance of 33.3%). Therefore, we concluded our GMM sufficient sensitive to detect clusters that correspond to categories in real BOLD data if, in fact, clear, discoverable categories exist in the data. Additionally, the use of BIC for model order selection, which considers the likelihood of the model and a penalty term for the number of parameters, ensured that we had a sufficient sample to discover clusters. BIC is valid when sample sizes are much larger than the number of parameters in the model^61^, and in our case, each subject had 180 trials, which is two orders of magnitude larger than the maximum number of clusters (3) in the data.

We then applied the same methods to estimate the GMM clusters that best described the actual BOLD data from the experiment. The results revealed variability in the number of clusters that best described each participant’s BOLD data (Fig. 1b). Furthermore, the clusters were heterogeneous, comprised of combinations of trials labeled as belonging to fear, happiness, and sadness categories, with no clear correspondence between the discovered clusters and the trial labels (Fig. 1c). We also examined the valence and arousal features of the scenarios within each cluster (available from the original dataset) but again observed no clear correspondence between these features and the clusters (Supplementary Fig. 2). Although we were unable to determine the features that the clusters were reflecting, our validation procedure with synthetic data, especially at low SNR, suggests that it is unlikely that the lack of correspondence between category labels and the clusters we discovered was due to insensitivity of the unsupervised method to detect fine-grained features in the stimuli.

We repeated the same supervised classification and unsupervised clustering analyses using BOLD data from 3 s post-stimulus intervals after each auditory scenario was finished, but before participants rated their experience of valence or arousal. During these post-stimulus intervals, participants were instructed to continue immersing themselves in the scenario. Results from these analyses were highly similar to those that used the 9 s immersion period. Specifically, we observed above-chance classification accuracy in the supervised analysis. Chance level was defined as one divided by the number of categories, or 1/3 (33.3%), and the mean accuracy was compared to chance performance using a one-sample t-test. The classifier achieved a statistically significant above-chance mean within-participant accuracy of 47.78% ($t(15) = 25.01$, $p<0.01$; Supplementary Fig. 3a). The number of estimated clusters from the unsupervised analysis varied across participants, and clusters contained a mixture of trials from each of the three labeled emotion categories (fear, happiness, sadness; Supplementary Fig. 3b,c). The valence and arousal features of trials were also mixed within each cluster (Supplementary Fig. 4). More details on this analysis are provided in the Supplementary Materials.

### Example 2: autonomic nervous system data from Hoemann et al.^52^

In our second comparison of supervised vs. unsupervised approaches, we examined the results of a study with 46 participants who freely labeled their emotions throughout their daily life experiences while ambulatory peripheral physiological signals were recorded. It is important to note in this context that in a majority of studies, experimenters label stimuli as belonging to a specific emotion category and assume that participants would categorize the stimuli in the same way. For example, a video clip of a puppy frolicking in a park is likely to be labeled with the folk category “happiness” (i.e., is thought to induce an instance of “happiness” in all participants), yet some participants may not experience happiness when viewing this video clip (e.g., those who have just experienced a death of a pet or those who have a fear of dogs). In contrast, this study provides a useful examination of participant-, rather than experimenter-, generated labels. The participant-generated emotion labels captured variation in the categories participants used to describe affective experiences. These labels included but were not limited to the traditional folk categories of Western psychology, but they refer to categories of states that often involve intense experiences of affect (i.e., of pleasure/displeasure and arousal), two basic features that are used to characterize emotions (e.g.,^19,62^). Additionally, the labels were generated from an explicit prompt that asked participants to list an emotions they were feeling. In the words of William James, “there is no limit to the number of possible different emotions which may exist, and why the emotions of different individuals may vary indefinitely, both as to their constitution and as to objects which call them forth” (^1^, pg. 454).

For 14 days over a 3-week period, participants’ electrocardiogram (ECG), impedance cardiogram (ICG), and electrodermal activity (EDA) were measured, along with their bodily movement and posture. Hoemann et al.^52^ used a novel biological-triggering approach to experience sampling, where participants were prompted to label their experience via a mobile phone app when they exhibited a pronounced and sustained change in interbeat interval (i.e., the time between successive heart beats) in the absence of physical movement. During each sampling event, participants freely labeled their current emotional experience and provided valence and arousal ratings on a 100-point sliding scale from − 50 (very unpleasant/deactivated) to 50 (very pleasant/activated). Physiology data from 60 s windows (30 s before and after a change in interbeat interval), were analyzed. EDA data were excluded from analyses due to difficulty differentiating these signals from artifacts and because EDA changes typically occur on a slower time scale than was used in the present analyses. It was also possible to derive respiration rate (RR) from the data using the ICG signal and impedance pneumography (e.g.,^63,64^), but we chose not to derive this measure because it is unlikely that meaningful RR changes would’ve occurred within the window of measurement. Six cardiovascular measures were used in the analysis at each sampling event, as detailed in Table 2. For more detail on the study design and available data, see the “Methods” section.

**Table 2:** Cardiovascular measures derived from peripheral physiological data.

Feature	Definition	Interpretation
Interbeat interval (IBI)	Time (in ms) between heartbeats (inverse of heart rate)	IBI describes how fast the heart is beating; greater IBI values indicate slower heart rate
Respiratory sinus arrhythmia (RSA)	High frequency variability in IBI which occurs within the respiratory frequency	RSA is an estimate of parasympathetic (PNS) influence on the heart; greater RSA values indicate PNS activation
Pre-ejection period (PEP)	Time (in ms) between the beginning of electrical stimulation of the heart and the opening of the aortic valve	PEP is an inverse estimate of cardiac contractility and sympathetic (SNS) control of the heart; greater PEP values indicate reduced contractility and SNS withdrawal
Left ventricular ejection time (LVET)	Time (in ms) between the opening and closing of the aortic valve	LVET describes how long it takes the heart to pump out blood; greater LVET values are associated with greater blood volume
Stroke volume (SV)	Volume (in mL) of blood ejected by the heart with each beat	SV describes blood flow; greater SV values indicate greater blood flow per heartbeat
Cardiac output (CO)	Volume (in L) of blood circulated in the body per unit of time (m)	CO describes blood flow over time; greater CO values indicate greater blood flow

Table adapted with permission from^52^.

**Figure 2:**
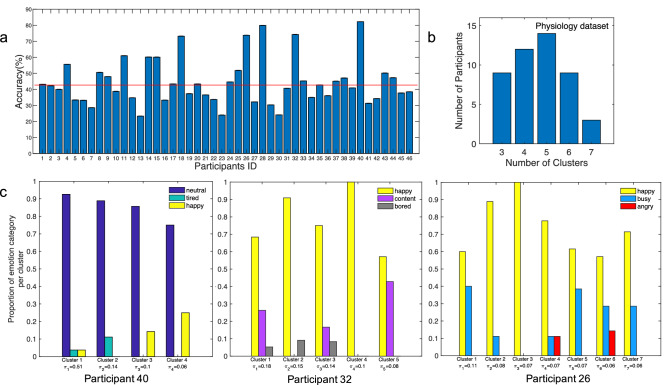
[ANS Data] Results from supervised and unsupervised analyses of ANS data. (**a**) Mean ± SEM classification accuracy for the top three emotion words used per participant. The red line represents the mean of the null distribution for chance performance of the group. (**b**) Distribution of participants by number of discovered physiology clusters resulting from DP-GMM. (**c**) Correspondence between the discovered clusters and the top three participant-specific emotion categories for three representative example participants with four, five, and seven clusters respectively (corresponding to Participant IDs 40, 32, and 26 in part a, with respective classification accuracy levels of 82.26%, 74.27%, and 73.80%). Bars represent the proportion of participant-generated emotion category per cluster. The total proportion of categories in each cluster sums to 1. The total number of emotion category labels used by example participants 40, 32, and 26 were 26, 17, and 9, respectively. The mixing proportion $\pi _k$ reported below each cluster is the probability that an observation comes from that cluster, which is representative of the size of the cluster.

We first conducted a supervised analysis of this dataset using each participant’s three most commonly used emotion words as the labels, because the emotion labels were subject-specific and variable in number, and we only classified data from corresponding events. An average of 72.74 (28.46) events were used for classification per subject across all three labels, with a minimum of 33 and a maximum of 158 events. To classify each participant’s physiology data based on their top three most commonly used emotion words, we trained and tuned a fully-connected neural network using fivefold cross-validation, dividing each participant’s data into 5 groups and iterating across all combinations of training on 4/5ths of the data and testing on the left-out 1/5th. Mean within-participant accuracy was calculated by averaging the accuracy across all cross-validation folds. Chance accuracy for this dataset could not be fairly defined as the reciprocal of the number of categories, because the number of events per label varied for each participant. We were unable to use oversampling methods^65^ to mitigate imbalances across categories (i.e., class imbalances) given the limited number of samples (an average of 72.74 per participant). We instead evaluated statistical significance of the mean accuracy across participants using a permutation test that maintained the imbalances. Specifically, we re-ran the classification 1000 times for each participant using shuffled training set labels. For each iteration of the permutation test we averaged the accuracy across participants, resulting in a distribution of 1000 mean values for chance-level performance (chance distribution shown in Supplementary Fig. 7). Our observed mean accuracy across-participants (47.1%) fell within the 5% tail of the chance distribution, indicating statistically significant above-chance accuracy of our classifier at detecting whether a participant’s data was labeled according to one of their top three most commonly used emotion words. The per subject accuracy plot is shown in Fig. 2.

The results of our supervised analysis were substantially different from those which resulted from an unsupervised analysis reported in^52^. The unsupervised analysis used a variant of Gaussian Mixture Modeling called Dirichlet Process Gaussian Mixture Modeling (DP-GMM^55,66^) to discover the number of clusters in the ANS data for each participant. The researchers found variable numbers of clusters for each participant, with a many-to-many correspondence between participant-derived emotion labels and discovered clusters in the data (Fig. 2). Participants’ valence and arousal ratings also varied substantially within and across the clusters they found.

### Example 3: self-reports of experience from Cowen and Keltner^9^

In our final comparison of supervised vs. unsupervised approaches, we re-analyzed self-report ratings of experience from^9^, in which 853 participants provided ratings of 2185 evocative video clips. A subsample of participants labeled their emotional experience after viewing the film clips by choosing from among a set of 34 emotion words provided by the experimenters; each participant viewed 30 clips and made categorical (yes/no) ratings of the 34 emotion words for each clip, where a value of 0 indicated a participant was not experiencing an instance of that emotion category during the clip, and a value of 1 reflected that a participant was experiencing an instance of that emotion category while viewing the clip. A different subsample of participants rated their affective experiences; each participant in this subsample viewed 12 video clips and rated them along 14 affective dimensions such as valence, arousal, effort, safety, etc., on a nine-point bipolar Likert scale (scale end-point labels were specific to each dimension, but 5 was always anchored at neutral).

**Figure 3:**
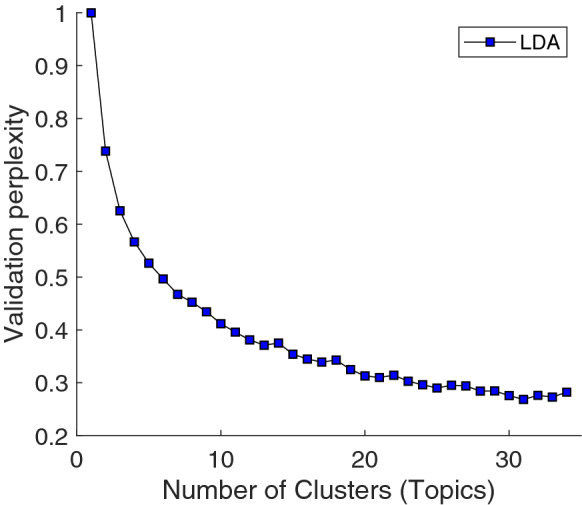
[Self-Report] Perplexity plot to discover the number of topics in LDA of emotion category self-report data. The x-axis represents the number of topics (discovered categories) and the y-axis represents the perplexity validation. There is no clear minimum value across the 34 categories, preventing us from identifying a clear clustering solution.

A traditional supervised analysis was not possible for this data set because the category labels that would be used for supervision would have to come from participants’ ratings. To contrast the SH-CCA reported in^9^ (see Supplementary Materials for an overview of their methods), we analyzed these data using an unsupervised clustering approach for the average yes/no emotion ratings of the 34 emotion categories across the 2185 film clips. A GMM was not appropriate to cluster these data because the emotion category ratings were not continuous. Instead, we applied Latent Dirichlet Allocation (LDA^67^) to discover clusters in the data. LDA is similar to GMM in that it is a mixture model, but LDA works on a collection of discrete data. It was originally developed for text corpora to discover a set of topics across a collection of documents in an unsupervised fashion. We also used LDA in this setting to perform statistical “topic modeling”, in a different sense; that is, to discover the number of abstract “topics” or emotion categories (i.e., discovered clusters) occurring across all ratings of video clips. Technically, each “topic” is represented as a different probability distribution over the 34 rated emotion categories. In LDA, a perplexity plot^67^ is used to evaluate the goodness-of-fit of the model given a fixed number of topics; its minimum indicates a suitable number of topics that optimizes the model’s performance. Our perplexity plot for the ratings of these video clips showed no clear minimum value (i.e., no statistically significant minimum value; see Supplementary Materials for details) across the 34 emotion categories, indicating that the 34 emotion categories could not be reliably reduced to a smaller number of separable clusters (i.e., no unique solution was possible; Fig. 3). In other words, the LDA analysis suggested that it was not possible to discover clear ‘clusters’ (i.e., lower dimensional representation) in these data. As a consequence, it was not possible to examine the distribution of affective features within clusters. Our finding of no lower dimensional representation using LDA was inconsistent with the conclusion of 24 to 26 categories in^9^, as well as a principal components analysis of the emotion category ratings (Supplementary Fig. 6) in which a scree plot followed by a parallel analysis^68^ indicated that only five clusters best explain systematic variance, while other ways of determining the PCA trunctation returned either larger values or even no truncation (consistent with the LDA analysis). In the absence of any clear consensus, one might decide that indeed there is no unambiguous structure in the data.

Keeping in the spirit of our first two analyses, where emotion categories were used as labels applied to BOLD and ANS data, we also approached the self-report data with a second analytic strategy. In this analysis, we used participants’ categorical emotion word ratings as the labels for the supervised analysis and the 14-dimensional vectors of mean affective ratings for the same clips as the inputs to our classification and clustering analyses. According to^9^, “75% of the videos elicited significant concordance for at least one category of emotion across raters [false discovery rate (FDR) $<0.05$], with concordance averaging 54% (chance level being 27%, obtained from simulated raters choosing randomly with the same base rates of category judgment observed in our data)” (pg. 3). However, when we analyzed the data we discovered that a smaller percentage of film clips could be unambiguously assigned to a single category. We assigned a label to each clip according to the highest rated emotion category for that video (e.g., if Clip 1 had a mean rating of 0.7 for sadness, 0.4 for fear, and 0.2 for anger, it was assigned the label sadness). Then, to ensure we had a sufficient number of samples for classification, we analyzed only data labeled as an emotion category that was assigned to at least 2.9% of the videos (because 34 emotion words were rated, we chose a threshold of 1/34, or 2.9%). Using this criterion, we observed only 41.6% of the videos elicited concordance with a single folk emotion category. Out of the 34 emotion categories, nine categories were the highest rated category for more than 2.9% of videos (adoration, aesthetic appreciation, anxiety, awe, disgust, fear, nostalgia, romance, sexual desire). Thus we classified videos labeled with these nine categories (908 video clips out of 2185).

**Figure 4:**
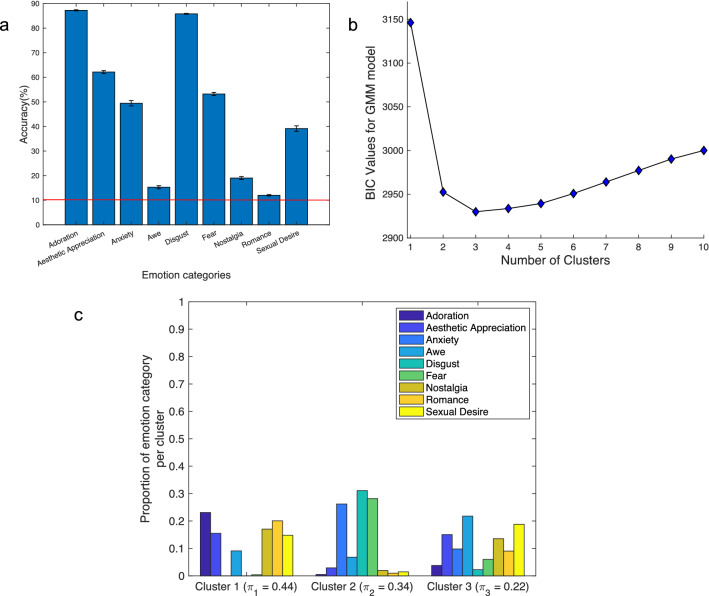
[Self-Report] Results from supervised and unsupervised analyses of self-report data. (**a**) Mean ± SEM classification accuracy for the most dominant six emotion category labels. The red line represents chance level accuracy (11.11%). (**b**) Plot of the BIC criterion used to discover the number of clusters in the GMM model. The x-axis represents the number of clusters to use and the y-axis represents the BIC value; the minimum value of BIC is the one chosen by the BIC criterion (here the minimum is 3 clusters). (**c**) Correspondence between clusters and the emotion category labels. Bars represent the proportion of trials from the dominant nine emotion categories within each discovered cluster. The total proportion of categories in each cluster sums to 1. The mixing proportion $\pi _k$ reported for each cluster is the probability that an observation comes from that cluster, which is representative of the size of the cluster.

We then conducted a supervised classification of the 14 dimensional vectors of affective features for each video clip, using the nine emotion categories as labels for the corresponding videos. Specifically, we trained and tuned a neural network^55^ using eightfold cross-validation, splitting the data into 8 groups and iterating across all combinations of training on 7/8ths of the data and testing on 1/8th of the data, to test whether a classifier could accurately detect the assigned video clip label at a rate above chance. Mean within-category accuracy was calculated by averaging the accuracy across all cross-validation folds. Chance level was defined as the reciprocal of the number of categories, or 1/9 (11.11%), and the mean across-category accuracy was compared to chance performance using a one-sample t-test. The classifier achieved a statistically significant above-chance mean across-category accuracy of 47.04% ($t(8) = 3.79$, $p<0.01$; Fig. 4a).

We then examined the correspondence between participant-defined folk category analysis of affective features to an unsupervised analysis of affective features using GMM. A GMM was appropriate for this dataset because the 14 dimensional vectors of affective features are continuous variables that can be modeled with a Gaussian mean and variance. Again using the Bayesian Information Criteria (BIC) to discover the number of clusters, we found a three cluster solution of videos (see BIC curve in Fig. 4b). After estimating these three clusters in our data using a GMM, we examined the correspondence between the clustering and the emotion category labels of the videos within each cluster. The discovered clusters were comprised of a mixture of videos labeled with the nine emotion categories. For example, Cluster 1 was comprised of a mixture of videos labeled as adoration, aesthetic appreciation, awe, nostalgia, romance, and sexual desire, 6 out of the 9 available labels (Fig. 4c).

## Discussion

We critically examined the use of emotion category labels in machine learning analyses across three studies, each sampling a different type of experiment and domain of data. In all three cases we contrasted the results of classification analyses supervised by labels with those from unsupervised approaches and observed that the methods did not produce concordant results. We obtained above-chance classification accuracy in the supervised analyses of all three datasets, suggesting that there is some information related to some of the emotion category labels. When analyzing these same datasets using unsupervised approaches, however, the clusters we discovered did not consistently correspond with the emotion category labels in any of the three cases. Validation of our analytical procedures with synthetic fMRI data demonstrated that the clusters discovered by unsupervised analyses in that case were reliable, and therefore explainable in some way. However, we were unable to determine the simple, easily-nameable features that the clusters were reflecting because the datasets tested were not designed for this purpose.

There are two possible explanations for this outcome that are worth emphasizing, either of which suggest extreme caution when interpreting the results of machine learning analyses to test psychological hypotheses. First, it is possible that the emotion category labels used in these three datasets (and most past studies of emotion categories) veridically reflect biological and psychological categories that exist in nature and are stable across contexts and individuals, and that latent emotion constructs do in fact, produce distinct, if graded, responses in brain, body, and behavior. If this possibility is correct, then we would expect above-chance accuracy for supervised classification because there is some reliable signal in the data that can be classified. Unsupervised analysis of this same data could result in clusters that meaningfully correspond to the labels, but could also result in clusters that do not correspond to the labels in any meaningful way because unsupervised clustering is completely dependent on the measurements taken. The inability of unsupervised methods to detect meaningful structure in the data could be due to an issue with the measurements. Specifically, if the data are too sparse, contain too much noise from other sources, or have undergone too much data reduction, the models may not be able to detect the structure in the data that meaningfully corresponds to emotion categories. In this situation, the discovered clusters are still reliable and therefore describing meaningful structure, but the structure is not reflective of emotion categories. For example, it is possible that the unsupervised clustering did not pick up on the signal in the measurements related to emotion categories if those signals are small compared to other signals that were unintended or not of interest to the experimenter; in this case, the clustering would be based on characteristics that are epiphenomenal to emotion (i.e., correlated features in the data that do not necessarily have anything to do with the ground truth labeling, such as time of day, what a participant had for breakfast, etc).

There is a second possible explanation for our findings, one that was originally suggested by William James and a growing number of modern scientists and philosophers: Western folk emotion categories may not be equally useful for making sense of the biological signals. There may be substantial contextual differences, individual differences, and cultural differences in how a human brain makes biological signals meaningful when creating emotional events to guide action. Past studies have observed substantial variation in the biological and psychological features of emotion categories, both within and across participants^42,43,52^, and across cultures^69–71^, suggesting that emotion categories are better thought of as populations of highly variable, context-specific instances^72,73^. A populations view of emotion categories draws on Darwin’s population thinking, in which a biological category, such as a species, consists of a population of variable individuals (^74^; for a discussion, see^42,72,73^). In a populations view, the magnitude of variation far exceeds the amount proposed in a classical or prototype view. A populations view treats variation among individual instances within a category as real and meaningful, and posits that the prototype of a biological category, as a single representation, is an abstract, statistical summary that need not exist in nature (for an extended discussion, see^42,75^). If this possibility is correct, then it would still be possible to achieve above-chance classification using a supervised approach, due to other commonalities in the data that the classifier could use to differentiate the data. Several studies have attempted to overcome this concern by constructing models that generalize across different types of stimuli (e.g., emotion induction with movie clips vs. music) within the same participants (e.g.,^24,26^) or the same stimuli across different individuals (e.g.,^24^). Nonetheless, these published studies were classifying a restricted range of emotional instances in each category. They did not sample the full range of heterogeneous instances of emotion category that have been observed in everyday life (as discussed in^32^). Instead, they cultivated a relatively small number of “stereotypical” instances per category. This is not an unreasonable observation given that most studies on emotion use stimulus sets that are curated using scientists’ intuitions about the nature of emotion categories that may therefore fail to capture the full range of variation in real world emotional responses. Furthermore, most studies do not attempt to generalize across both stimulus type and participants at the same time, the notable exception being a classification study performed on a meta-analytic database^32^). This study, which classified activation maps from different studies, across stimulus types and induction methods, noted systematic differences in how experimenters induced different emotion categories across studies; and indeed, methodological variables such as stimulus type and method of induction were classified above chance levels. Consistent with these observations, the first data set we examined (^51^ contained more stimulus variation than is typical for most published studies using machine learning (see Table 1 for the number of unique stimuli per category that is typical of past studies), and the second data set^52^ used an objective, empirical criterion for sampling stimulus events. Taken together, our results may suggest the possibility that supervised machine learning is capable of discovering signal that is stipulated by the stimuli used to manipulate emotion, but that signal may not be sufficiently strong to be detected with unsupervised analyses alongside other real world variability in other features. This makes the accuracy of the labels critical, since they impose the viewpoint from which that structure is discovered, and thus suggests the need for careful, cross-study, skeptical interpretation of supervised results until validity of the labels can be scientifically determined.

### Implications for future research

The present findings do not allow us to discern which of these two interpretations is correct, but they do highlight several important implications and course corrections for future research. First, there is increased burden on researchers using supervised learning with category labels to think about and empirically explore the validity of their labels, and potential constraints in their stimulus sets and methods, rather than assuming that the labels are the ground truth and then sampling with that assumed prototype or stereotype in mind. Studies that ask participants to report on their emotional experiences find wide variation in the number and granularity of their emotion categories^72,76^, and consequently, the emotion labels typically used in supervised classification analyses, which correspond to Western folk emotion categories, may not accurately encompass the repertoire of biological categories that describe all individuals equally well in all situations and therefore fail to provide the best mapping to biological data (for a discussion, see^14^). It is also important to consider the possibility that instances of emotion might be categorized in multiple ways, simultaneously, which is often referred to as a ‘mixed’ emotion (as discussed in^77^). Future studies should consider combinations of emotion words as descriptors of emotional instances to better capture the complexity of emotional experiences.

To cultivate a robust and replicable science, researchers must design studies in a way that allows for an explicit test of whether the category labels they apply are the best way to estimate the structure in their data. Specifically, it would be optimal to sample many domains of features at higher dimensionality. This would include sampling biological and psychological features in the same study in a temporally sensitive way. An ideal study would measure a person’s internal context (e.g., the person’s metabolic condition, the past experiences that come to mind) and external context (e.g., whether a person is at work, school, or home, who else is present, broader cultural conditions). In addition to measuring internal and external context, an ideal study would also measure a broad set of mental features that might describe an instance of emotion, such as appraisal features (how the situation is experienced; e.g., as novel or familiar, requiring effort, or conducive to one’s goals, etc^78,79^) and functional features (the goals that a person is attempting to meet during the instance; e.g., to avoid a predator, to feel closer to someone, to win a competition, to gain social approval, etc^80,81^, all of which vary in dynamic ways over time. Combining these measures with an objective, empirical way of sampling stimulus events (as in^52^) would provide an optimal way to accurately capture and interpret meaningful structure in machine learning analyses of emotion.

Additionally, future researchers should pay special attention to variable patterns of measurements across individuals or studies associated with the same labels, especially if these differing patterns result from classification using similar stimuli and/or methods. At the very least, researchers must start using more than one machine learning method to analyze their data to discover the extent to which their modeling methods are responsible for any observed inconsistencies. We recommend that researchers routinely compare supervised and unsupervised approaches on their data and report both or at least use multiple supervised classification algorithms or feature selection methods on the same dataset to explore implications of methodological decisions on their solutions.

Finally, we echo recent suggestions that as a field, we must substantially increase the power of future studies^82–85^. Increasing the number of participants is not enough—the number of unique, variable stimuli per category is also important. Gonzalez-Castillo et al.^86^ demonstrated that scanning a smaller number of subjects for a long duration reveals patterns of whole-brain BOLD responses that are not seen in traditional neuroimaging studies when a large number of subjects are scanned for only a short duration^86^. Increased within-subject power will make it more likely that studies will either find something meaningful with unsupervised methods or to find more consistent results with a supervised approach. Most current datasets have too few instances and low signal, which does not allow researchers to rule out sparsity or noise as the reason for a lack of identifiable structure when using unsupervised clustering. Finally, if studies are designed with the possibility of discovering new categories (rather than merely confirming the existence of an experimenter’s preferred folk categories), and those studies are adequately powered to detect reliable clusters that explain more variance than the categories, then researchers will be better positioned to discover new mental categories to be studied in more depth.

## Conclusion

The present study highlights the importance of questioning assumptions and the validity of using folk labels in the study of psychological categories. Our findings of inconsistency between supervised and unsupervised approaches can reflect two possibilities: (1) category labels in the study of emotion may reflect biological categories that exist in nature, and the observed inconsistency is due to measurement error or methodological considerations, or (2) these category labels are not useful as biological kinds that are stable across individuals and contexts. Instead, these words may refer to populations of highly variable, context-specific instances. The goal of the present study was not to demonstrate which of these possibilities is correct, but rather, to suggest that the variability observed in past studies of emotion classification may be reflecting a real discovery that should not be dismissed as error. Rather than claiming a particular truth, we are highlighting the fact that the current analytic strategy of using folk psychology categories to guide study development and data analysis should be considered. While we used the domain of emotion as a test case to highlight the importance of questioning category labels, the implications of our findings and suggestions for future research apply to research on psychological categories across a variety of domains, and may ultimately provide a way forward in identifying mental categories that more cleanly map to measurements of brain activity, physiological changes in the body, and behavior.

## Methods

### Dataset 1: fMRI BOLD data

#### Data overview

We used data previously reported in^51^, which includes comprehensive study design details. Briefly, 16 participants (8 female, ages 19 to 30 years) participated in the study. Participants completed a series of two training sessions, one 24–48 h prior to the scan and the second immediately before the fMRI scan, in which they listened to auditory scenarios intended to induce the emotion categories of fear, sadness, and happiness. During the scan participants heard a shorter, core form of each scenario during a 9 s trial and were instructed to fully immerse themselves mentally in each scenario as they listened. Following each 9 s trial, there was a 3 s post-stimulus interval during which participants continued immersing themselves in the scenario. Participants heard 30 unique scenarios for each of the three folk emotion categories. Each scenario was presented twice, resulting in a total of 180 trials. Given the assumption of past emotion classification studies (e.g.,^24,26,29,30^) that all trials for a given emotion category should share the same pattern of neural activation, we included all repeating trials in our analyses. In other words, we assumed that stimuli would induce the same brain response on each presentation. Scenarios varied in valence and arousal, with some scenarios intended to evoke typical valence/emotion combinations (i.e., pleasant happiness) and others intended to induce atypical combinations (i.e., unpleasant happiness). All subjects provided informed consent, and all recruitment procedures and experimental protocols were approved by the Institutional Review Board of the original study’s institution (Emory University Institutional Review Board). All methods were carried out in accordance with relevant guidelines and regulations.

The supervised classification and unsupervised clustering analyses explained below were conducted twice, in an identical manner. Analyses were first conducted on whole-brain beta maps from the 9 s scenario immersion window of each trial, and then repeated on whole-brain beta maps from the 3 s post-stimulus interval after the auditory scenario was presented, while participants were still immersing themselves in the scenario.

#### Preprocessing

fMRI preprocessing steps were conducted in AFNI^87^ and were identical to those in^51^: slice time correction, motion correction, spatial smoothing (6 mm FWHM Gaussian kernel), and percent signal change normalization for each run. Spatial smoothing has not typically been performed in published MVPA studies of emotion. The issue of spatial smoothing in MVPA studies is complex, however, and the utility of smoothing depends on the data and question at hand^88^. In the absence of functional alignment across individuals, we expect that signals useful for clustering and classification at this spatial scale will be relatively low frequency and that smoothing will improve performance.

Data from each 9 s trial was then convolved with a canonical hemodynamic response function to generate a single “beta” value for each trial, resulting in 60 “beta maps” each for the nominal emotion categories of fear, sadness, and happiness. Beta maps were also generated for the 3 s post-stimulus interval for each trial using this same method.

#### Supervised classification

To test whether a supervised classification algorithm could detect distinguishable patterns of neural activity for emotion categories, we carried out within-subject classification using a 3D convolutional neural network (CNN; additional details of the neural network configuration are in Supplementary Materials). This analysis was conducted using the deep learning toolbox in MATLAB (https://www.mathworks.com/products/deep-learning.html). The 3D CNN was used to classify data as belonging to one of three possible conditions: happiness, sadness, or fear. Classification was conducted on whole-brain data, and the neural network was used to automatically perform feature selection, as is common in many image classification reports^89^. Specifically, dropout layers were used on the input feature layer as well as subsequent hidden layers to optimize the automatic feature selection procedure and regularize the neural network weights. The classifier was trained using a leave-one-run-out procedure where training was performed on BOLD data from 5 runs and testing was applied to the data from the remaining 6th run. Cross-validation was performed across all iterations of leaving-one-run-out and accuracy was calculated as an average percentage of correct classification across all cross-validation runs. Chance level performance was defined as the reciprocal of the number of categories, or 33.3%. A one-sample t-test against chance level accuracy was conducted to determine whether mean classifier performance across participants was significantly above chance.

#### Unsupervised clustering

Our unsupervised clustering approach used a combination of dimension reduction and Gaussian Mixture Modeling, as described next.

##### Principal component analysis (PCA)

We applied PCA to the high-dimensional (total number of fMRI voxels) beta maps. Dimension reduction was necessary to enable reliable cluster estimation given the limited number of data samples available. Statistical assumptions that underlie the choice of PCA for this purpose are described in Supplementary Materials but we note here that the number of principal components used was determined jointly with the number of Gaussian Mixture Model clusters identified, using the Bayesian Information Criterion (BIC, see Supplementary Materials for details). The result of the PCA was a feature vector $\alpha _t$ for each trial for each participant.

##### Probabilistic graphical model

**Figure 5:**
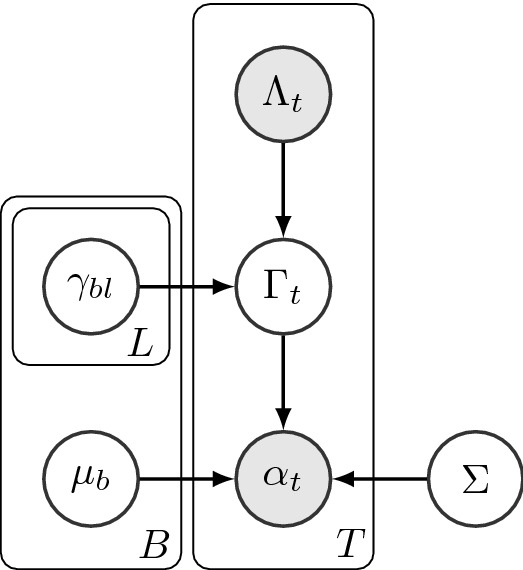
[BOLD Data] Probabilistic graphical model for GMM analysis.

To replace the assumption that the labels are known, we applied an unsupervised generative probability model for automatically discovering brain labels from the data $\alpha _t$. The probabilistic graphical model (PGM) that describes our probabilistic dependence/independence assumptions is shown below (Fig. 5). Circles indicate random variables and the statistical dependence between pairs of random variables is shown by directed arrows (graph edges). Shaded circles indicate that the random variable is observed, while clear circles indicate that it is unobserved. Rectangles show repetitions of each random variable (e.g. over emotion category or run) with the number of repetitions shown in the right bottom corner of the rectangle.

The input features to the PGM were the low-dimensional representation of beta maps, the random vectors $\alpha _t$, with dimension *D* equal to the number of retained principal components obtained from PCA for each trial *t* and for each participant (we do not use additional indices for each participant to simplify the notation). There are a total of *T* trials per participant, each with an unknown brain label $\Gamma _t$ to be estimated and an emotion label $\Lambda _t$. The key innovation here is this distinction; while $\Lambda _t$ is known, supplied by the experiment designer, the $\Gamma _t$’s are unknown and latent. We assume that each value of $\Gamma$ depends on both the associated $\Lambda$ and a latent “mixing coefficient” $\gamma _{bl}$, indexed by both cluster label *b* and emotion index *l*. For each participant, there is a mixture of *B* Gaussian distributions, or soft clusters, each of which is associated to a brain label. The Gaussian distribution for brain label *b* has a mean $\mu _b$ and a covariance matrix $\Sigma$ shared across the brain labels. In other words, the mixture of *B* Gaussian distributions models all the trials for each participant (i.e., the total of *T* trials are soft-clustered into *B* clusters, each of which is represented by a distinct Gaussian distributions). The GMM mixing proportions for each participant’s $\gamma _{bl}$ is the prior probability that the given beta map comes from the *b*th cluster given label *l*.

##### Gaussian mixture modeling

Based on the model shown in the PGM, we estimated the parameters of a GMM for each participant across all trials and all emotion category labels. Given a vector $\alpha _t$ calculated by PCA, and an emotion category label *l*, the GMM has the form:

1 $$\begin{aligned} p(\alpha _t\mid \Lambda _t= l)=\sum _{b=1}^{B}\gamma _{bl}~ g(\alpha _t\mid \mu _b,\Sigma ). \end{aligned}$$

where *b* represents the labels of underlying brain activity, $b= 1,2,\ldots,B$, $\Lambda$ takes on the values of each a priori emotion labels, $\gamma _{bl}$ is the probability of observing brain label *b* given label *l*, and $mu_b$ and *Sigma* are the GMM model parameters. $\Sigma$ is taken to be diagonal (reasonable since we applied PCA first) and shared across brain labels. Maximum likelihood estimates of the GMM parameters are obtained using an iterative Expectation-Maximization algorithm (1). Note that *B* along with the number of PCA components (*D*, dimension of $\alpha$) are determined using the Bayesian Information Criteria (BIC) across a range of possible values for both (see Supplementary Materials for details).

### Dataset 2: autonomic nervous system data

#### Data overview

Here we report on analyses of data from a concurrent study^52^ in which ambulatory peripheral physiology and self-report data from each of 46 participants was collected for 14 days over a 3-week period. The signals acquired included electrocardiography (ECG), impedance cardiography (ICG), and electrodermal activity (EDA), along with participant’s bodily movement and posture. During the 2 week period, participants were prompted to respond to a survey via a smartphone device whenever their interbeat interval (IBI) exhibited a pronounced increase or decrease sustained over more than 8 s in the absence of any physical movement or posture change. The IBI-change threshold was tailored for each individual participant throughout the study to produce an appropriate number of survey prompts per day. The survey asked what participants were doing when they received the prompt and included a rating of their overall valence and arousal on a scale from − 50 to 50 (unpleasant to pleasant; low to high arousal), and free labeling of the emotions they were experiencing. Participants received an average of 9 prompts a day. All subjects provided informed consent, and all recruitment procedures and experimental protocols were approved by the Institutional Review Board of the original study’s institution (Northeastern University Institutional Review Board). All methods were carried out in accordance with relevant guidelines and regulations.

#### Preprocessing

Raw physiology data was preprocessed using the researchers’ in-house Python pipeline that involved signal dependent filtering, quality control checks and feature extraction. EDA data were excluded from the analyses due to issues with differentiating these signals from artifacts, as well as the slower time scale on which changes in EDA typically occur. As part of the initial processing, researchers calculated six measures: respiratory sinus arrhythmia (RSA), interbeat interval (IBI), pre-ejection period (PEPr), left ventricular ejection time (LVET), stroke volume (SV), and cardiac output (CO). For each of the six measures, at each event, they calculated a change score as a difference of means between the 30 second period before and after the change in interbeat interval that triggered the survey prompt. This resulted in a data vector of length 6 for each event, which was used as input in the analysis.

#### Supervised classification

Because both the number and value of emotion category labels were specific to each subject, we only classified events corresponding to the top three emotion words for each participant. On average, for a single participant, we classified 72.74 (28.46) events out of 122.87 (18.05) total events. We trained and tuned a fully-connected neural network using cross-validation, employing dropout layers to prevent overfitting. We ran within-subject classification using a fivefold cross validation approach. Because participants varied in the number of times they used each label, chance performance could not be defined as simply the reciprocal of the number of categories. Instead, we used a permutation test to evaluate statistical significance. Specifically, we ran 1000 permutations of shuffled training labels followed by classification, averaging the classification accuracies across all participants for every iteration of the permutation. This resulted in a null distribution comprised of 1000 group mean chance accuracies. We compared the actual group mean performance to the null distribution of chance performance obtained by this process.

#### Unsupervised clustering

To cluster the data vector of each event for each participant, the researchers who conducted this study^52^ used Dirichlet Process Gaussian Mixture Model (DP-GMM) to discover the number and membership of clusters for each participant. DP-GMM is an infinite mixture model^55,66^ with the Dirichlet Process as a prior distribution on the number of clusters. In practice, the approximate inference algorithm uses a truncated distribution, not the infinite one, with a fixed maximum number of clusters, but the number of clusters nonetheless depends on the data (see^52^ for more details). They used the Scikit-learn implementation of DP-GMM^90^, using number of events as the initial number of clusters for each participant. Full covariance type was used with Dirichlet Process weight concentration prior and mean of the data as the mean prior. Since the Scikit-learn implementation of DP-GMM tends to give slightly different results based on $random\_state$ parameter, the method was run 100 times and the solution with the highest lower bound value on the likelihood was kept.

### Dataset 3: self-report data

#### Data overview

Self-report data used in our analysis was taken from^9^. In that study, 853 participants provided ratings for 2185 film clips. A subset of participants provided ratings of 14 affective dimensions on a nine-point Likert scale. Each video clip was rated by nine participants, and we used the mean rating for each clip for each of the 14 affective dimensions as input to our analyses. A separate subset of participants made categorical (yes/no) ratings of 34 emotion categories. We used mean response data, where a value of 1 indicated that the given emotion category was chosen by all participants, and a value of 0 indicated that the category was never chosen. Each film was rated by 9 to 17 participants. All subjects provided informed consent, and all recruitment procedures and experimental protocols were approved by the Institutional Review Board of the original study’s institution (the Institutional Review Board at the University of California, Berkeley). All methods were carried out in accordance with relevant guidelines and regulations.

#### Latent Dirichlet Allocation

We conducted analyses on the mean response data for each video using a topic modeling approach based on Latent Dirichlet Allocation (LDA) to discover the abstract topics occurring in the collection of videos. In general, Latent Dirichlet Allocation (LDA) is a generative statistical model that allows sets of observations to be explained by latent topics that explain the similarity between parts of the data^67^. In LDA, the data from a text corpus of documents is analysed, where words in documents are used to determine both the distribution over topics and the distribution of topics over the documents. Thus in this case the assumption is that each subject’s response corresponds to an unknown number of unknown topics and the same is true for each clip In LDA, each data point is viewed as a mixture of various topics where each data point is considered to have a set of topics that are estimated by means of the LDA algorithm. The assumption typically made in LDA is that the topic distribution has a sparse Dirichlet prior, which is guided by the assumption that each data point only corresponds to a small set of topics and that each topics itself is a distributions over a small set of actual emotion categories. Or goal using LDA was to find a low-dimensional set of topics (so here discovered emotion categories) that could capture all the 34 emotion categories on which the clips were rated. Thus if low-dimensional structure was statistically justified in the ratings according to the LDA model, meaning that the emotion categories could be grouped into a smaller number of “emotion topics”, LDA is presumed to be able to discover that structure. The LDA model is shown as a probabilistic graphical model in Supplementary Figure 1. More detail on the LDA model is reported in Supplementary Materials.

#### Supervised classification

To test whether we could successfully classify video clips as belonging to specific emotion categories based on ratings of 14 affective features, we trained and tuned a fully connected neural network using cross-validation. First we labeled the video clips based on the highest rated category across subjects (i.e., we choose the category with the maximum mean rating as the label for each video clip). Then, to ensure we had enough instances for classification of each category, we chose only those categories that were used as labels for more than 2.9% of the total video clips, which is the percentage of data samples per emotion category if data was uniformly distributed among 34 emotion categories. In other words, we only classified categories that were chosen as the highest rated category for more than 67 out of 2185 clips. Only nine emotion categories met these criteria: adoration, aesthetic appreciation, anxiety, awe, disgust, fear, nostalgia, romance, sexual desire, resulting in classification of 980 videos. The classifier was trained with a sixfold cross validation approach; to impose a more balanced class distribution we over-sampled the minority classes (i.e, the class with the least number of samples) to be equivalent in frequency to the majority class (i.e., the class with the most number of samples). The average accuracy was calculated as the percentage of correct classifications across all validation sets. Chance level performance was defined as the reciprocal of the number of categories, or 11.11%. A one-sample t-test against chance level accuracy was conducted to determine whether mean classifier performance across emotion categories was significantly above chance.

#### Unsupervised clustering

Similar to the fMRI analysis, here we adopted a GMM clustering technique to cluster the video clips based on their 14 dimensional affective features. Each of the 14 features is a mean rating across a subset of subjects, which varies from 1 to 9. The 14 dimensional affective features are, therefore, continuous bounded values in each dimension and can be fit reasonably well by a GMM. The number of cluster was determined using the BIC across a range of possible values for the the number of clusters, similar to what was done in Dataset 1.

## Supplementary information

Supplementary Information.

## Data Availability

Datasets 1 and 2 are available from the corresponding authors upon request. Dataset 3 is publicly available through the cited manuscript.

## Code availability

The code to analyze data is available from corresponding authors upon request.
