# Author Correction: Comparing supervised and unsupervised approaches to emotion categorization in the human brain, body, and subjective experience

**DOI:** 10.1038/s41598-021-89878-x

**Published:** 2021-05-10

**Authors:** Bahar Azari, Christiana Westlin, Ajay B. Satpute, J. Benjamin Hutchinson, Philip A. Kragel, Katie Hoemann, Zulqarnain Khan, Jolie B. Wormwood, Karen S. Quigley, Deniz Erdogmus, Jennifer Dy, Dana H. Brooks, Lisa Feldman Barrett

**Affiliations:** 1 Department of Electrical & Computer Engineering, College of Engineering, Northeastern University, Boston, MA USA; 2 Department of Psychology, College of Science, Northeastern University, Boston, MA USA; 3 Department of Psychology, University of Oregon, Eugene, OR USA; 4 Institute of Cognitive Science, University of Colorado Boulder, Boulder, CO USA; 5 Department of Psychology, University of New Hampshire, Durham, NH USA; 6 Edith Nourse Rogers Veterans Hospital, Bedford, MA USA; 7 Department of Psychiatry, Massachusetts General Hospital, Boston, MA USA; 8 Athinoula A. Martinos Center for Biomedical Imaging, Massachusetts General Hospital, Boston, MA USA

Correction to: *Scientific Reports* 10.1038/s41598-020-77117-8, published online 20 November 2020

The Acknowledgements section in this Article was omitted. The Acknowledgements section should read:

“Part of this work was supported by Army Research Institute grant W911NF-16-1-0191 (PIs: K. Quigley & J. Wormwood). The views, opinions, and/or findings contained in this report (paper) are those of the authors and shall not be construed as an official Department of the Army position, policy, or decision, unless so designated by other documents. This work was also supported by National Institute of Mental Health grant R01-MH113234 (PI: L. F. Barrett) and National Cancer Institute grant U01-CA193632 (PI: L.F. Barrett).”

