# Supplementary material for: Comparing supervised and unsupervised approaches to emotion categorization in the human brain, body, and subjective experience
Source: Sci Rep. 2020 Nov 20;10:20284. doi: 10.1038/s41598-020-77117-8 (PMC7679385; doi:10.1038/s41598-020-77117-8)
Supplement: Supplementary file 1 — Supplementary Information. [file 41598_2020_77117_MOESM1_ESM.pdf]

Supplementary Material

Supplementary Methods

All analyses were conducted in MATLAB version R2019a using custom code, unless otherwise mentioned.

Dataset 1: fMRI BOLD data

Convolutional Neural Network Details

The details of the 3d Convolutional Neural Network configuration are as follows:

- Three 3D convolutional layers with kernel size of 5 were used.
- After each convolutional layer, we used a batch normalization layer to speed up training and reduce sensitivity to network initialization.
- Beach (feature/hidden) layer we also use dropout layers to regularize the neural network weights and optimize the selected features, [8].
- A hyperbolic tangent activation layer was applied after batch normalization.
- Downsampling by a factor of 2 with stride 2 was performed using a max pooling layer on the activation outputs.
- Finally, a fully connected layer followed by a soft max produced the classification probabilities.
- In training, the batch size was 20, the maximum epoch was 10, and the learning rate was chosen as a hyperparameter to be equal 0.001.
- ADAM optimization algorithm, [6], was used to iteratively update network weights in the training procedure.

Joint Estimation of Principal Component and Cluster Number using BIC

We performed a grid search over both the value of D, the number of principle components to retain, and the values of B, number of clusters/mixture components. We evaluated the model for every combination of (B, D) in a reasonable range using the BIC. Specifically, the value for D was varied from 1 to 179 (one less than the number of trials $T = 180$) and is the maximum dimension of variance that can be captured with $T < V$ trials, where V is the number of voxels. The value for B was varied from 1 to 10. We chose the (B, D) pair from this range that yielded the minimum BIC.

Details of Model Performance Evaluation using Synthetic Data

As described in the main text, we generated synthetic data to test the ability of our clustering model to capture the signal induced by stimuli embedded in noise. We generated synthetic data using the neurosim MATLAB package (https://github.com/ContextLab/neurosim) with the generative process proposed in ([7]). The synthetic data was designed to imitate BOLD data with clear, discoverable categories. For a given signal-to-noise ratio (SNR), an experimental design matrix, and brain voxel location, the package generates a set of synthesized voxel activations. For each emotion category, we used 20 randomly chosen spherical regions in the brain with varying BOLD amplitude during each trial. Each of these regions was assigned a single radial basis function whose spatial center and width was chosen uniformly but restricted to remain within the limits of a standard human brain. The synthesized "brain image" for each trial t was a weighted combination of these 20 basis functions for the specific emotion category active during that trial. Zero-mean Gaussian noise was added as the last step to simulate the measurement noise. The standard deviation of the Gaussian noise was calculated using the SNR supplied. We varied the SNR in our study from 0.01 to 100 in the range of [0.01, 0.05, 0.1, 0.5, 1, 5, 10, 100] to assess the performance of our model under varying levels of noise. In a Monte Carlo simulation, for each SNR value, we performed 500 random realizations of synthesized brain images and calculated the accuracy of our model at clustering the emotion categories. We reported the average accuracy across all random realizations. To evaluate the supervised performance on the same synthetic data we used the same structure for the CNN as was used for the actual fMRI BOLD data, described above.

Dataset 2: Autonomic Nervous System Data

Neural Network Details

The Details of the neural network configuration are as follows:

- Three fully-connected layers of size 5, 4, 3 were used.
- After each fully-connected layer we used a batch normalization layer to speed up training of the network and reduce the sensitivity to network initialization.
- A rectified linear unit (ReLU) activation layer was applied after batch normalization.
- Finally a soft max layer generated the classification probabilities.
- The batch size was 10, the maximum epoch was set to 50, and the learning rate was chosen as a hyperparameter to be equal 0.001.

- ADAM optimization algorithm, Kingma and Ba [6], was used to iteratively update network weights in the training procedure.

Dataset 3: Self-Report Data

Overview of Cowen & Keltner 2017 split-half canonical correlation analysis

The experimenters did not provide emotion labels for the film clips, but the clips were chosen with those specific 34 labels in mind (from Cowen & Keltner, "The videos were gathered by querying search engines and content aggregation websites with contextual phrases targeting 34 emotion categories"; pg. 2 [2]). However, it can be debated whether the initial analyses reported in [2] were free from strong assumptions about the existence of certain emotion categories. The researchers devised a split-half canonical correlation analysis (SH-CCA) in which they correlated the mean emotion category ratings for each clip from half the subsample of participants with the mean ratings from the other half. They reported that the results of the SH-CCA indicated that the data contained between 24 and 26 categories of emotional experience. Because the categories in the SH-CCA were pre-specified rather than discovered (i.e.,one half of the categorical ratings constrained the analysis of data the other half), this method is more constrained than is optimal for a fully unsupervised analysis. Indeed, it might be argued that the categorical nature of the ratings and using the same labels for video selection and for participant ratings makes this SH-CCA more likely to reveal a solution consistent with a supervised classification than data-driven clustering ([1]).

Latent Dirichlet Allocation

The LDA model is shown as a probabilistic graphical model below. The interpretation of the symbols in the graph is the same as described in the Methods section for Dataset 1.

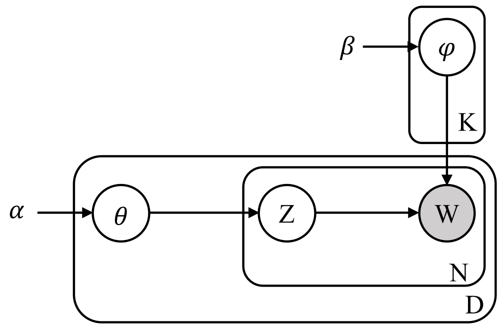

Supplementary Figure 1: **[Self-Report] Probabilistic graphical model for LDA analysis**

Specifically, we have a collection of D video clips, each of which is a mixture over K topics (i.e., video

clip $d \in \{1, ..., D\}$ has a associated K dimensional vector θ_d that shows its distribution over topics). Each topic k is characterized by 34-dimensional vectors ψ_k, which is a distribution over the predefined emotion categories. For each video clip, N is the total number of 'yes' answers for all of the pre-defined emotion categories, W is a categorical variable showing the 'yes' answer for a specific predefined emotion category, and Z is the corresponding topic index. Finally, α and β are the parameters of the Dirichlet priors for the topic mixtures θ and topics ϕ, respectively. The corresponding probabilistic generative model for this LDA model is as follows:

$$\theta \sim \text{Dirichlet}(\alpha),$$
$$\phi \sim \text{Dirichlet}(\beta),$$
$$Z \sim \text{Categorical}(\theta),$$
$$W \sim \text{Categorical}(\phi),$$

where the Dirichlet distribution is given by

$$p(\theta \mid \alpha) = \frac{1}{\mathbf{B}(\alpha)} \prod_{K}^{i=1} \theta_i^{\alpha_i - 1}$$

and $\mathbf{B}(\alpha)$ denotes the multivariate Beta function.

We adapted a collapsed Gibbs sampling method for LDA model based on [4] in MATLAB's Text Analytics Toolbox to solve for the unknown parameters of the model, including the topic distributions and the distribution over topic ϕ for each video clip, θ.

To decide on a suitable number of topics for LDA, we compared the goodness-of-fit of LDA models across varying numbers of topics. We evaluated the goodness-of-fit of an LDA model after by training on all videos except for a held-out subset by calculating the perplexity on the held-out subset:

$$\text{perplexity}(D_{test}) = \exp\left\{-\frac{\sum_{d=1}^{M} \log p\ (W \mid \alpha, \psi)}{MN}\right\} \tag{1}$$

where D_{test} is the held-out collection of M videos. The perplexity indicates how well the model described the data, with a lower perplexity suggesting a better fit. We used 10-fold cross-validation by randomly partitioning the original dataset into ten equal size subsets. Nine of the subsets were used for training, and the remaining subset for validation. The cross-validation process was then repeated 10 times for all folds and each of the 10 subsets was used exactly once as the validation set. The average perplexity across 10

folds was used for choosing the number of topics (see Fig. 3 in the main document).

Neural Network Details

The Details of the neural network configuration are as follows:

- Three fully-connected layers of size 10, 8, 6 were used.
- After each fully-connected layer we used a batch normalization layer to speed up training of the network and reduce the sensitivity to network initialization.
- A rectified linear unit (ReLU) activation layer was applied after batch normalization.
- Finally a soft max layer generated the classification probabilities.
- The batch size was 20, the maximum epoch was set to 10, and the learning rate was chosen as a hyperparameter to be equal 0.001.
- ADAM optimization algorithm, Kingma and Ba [6], was used to iteratively update network weights in the training procedure.

Permutation Test

To evaluate the statistical significance of our classification findings, we conducted a permutation test based on the definition from [3]. The details of the procedure are as follows. Given the original data set $D = \{(\mathbf{X}_i, y_i)\}_i^n$ and a permutation function for n elements, π, one can permute the labels y of data set D with the aim to produce a new data set $D' = \{(\mathbf{X}_i, \pi(y)_i)\}_i^n$ whose marginal distributions of features $p(\mathbf{X})$ and labels $p(y)$ are the same as those of the original data set while the dependence between features and labels are broken. The new data set D' is intuitively known to come from chance. Based on the definition by Good (2000), we defined $\mathcal{D}$ as a set of k randomized permutations D' of the original data set D sampled from a null distribution. We then calculated the p-value based on the following equation, where $a(f, D')$ is the training accuracy computed using the permuted labels in D':

$$p = \frac{|D' \in \mathcal{D} : a(f, D') \geq a(f, D)| + 1}{k + 1}, \tag{2}$$

The $|\cdot|$ represent the cardinality (size) of the set. The calculated p-value represents the fraction of permuted samples where the classifier's accuracy was better in the random, permuted data than the original data, reflecting the probability that the classification accuracy for our data was obtained by chance.

No Significant Minimum in Perplexity Plot (Fig. 3)

In Fig. 3, we cannot decide in favor of a specific value for the number of clusters because there is not a clear minimum. To confirm that there is no minimum attained that is statistically significant, we ran a paired t-test for each pair consisting of the smallest value at 31 and one of the adjacent values at 30 or 32. We used the perplexity value across 10 folds of validation as inputs in the t-test. According to the t-test result, the perplexity value at 31 was not significantly different from 30 or 32 ($p > 0.1$). Therefore, we cannot choose that as the minimum perplexity.

Supplementary Figures

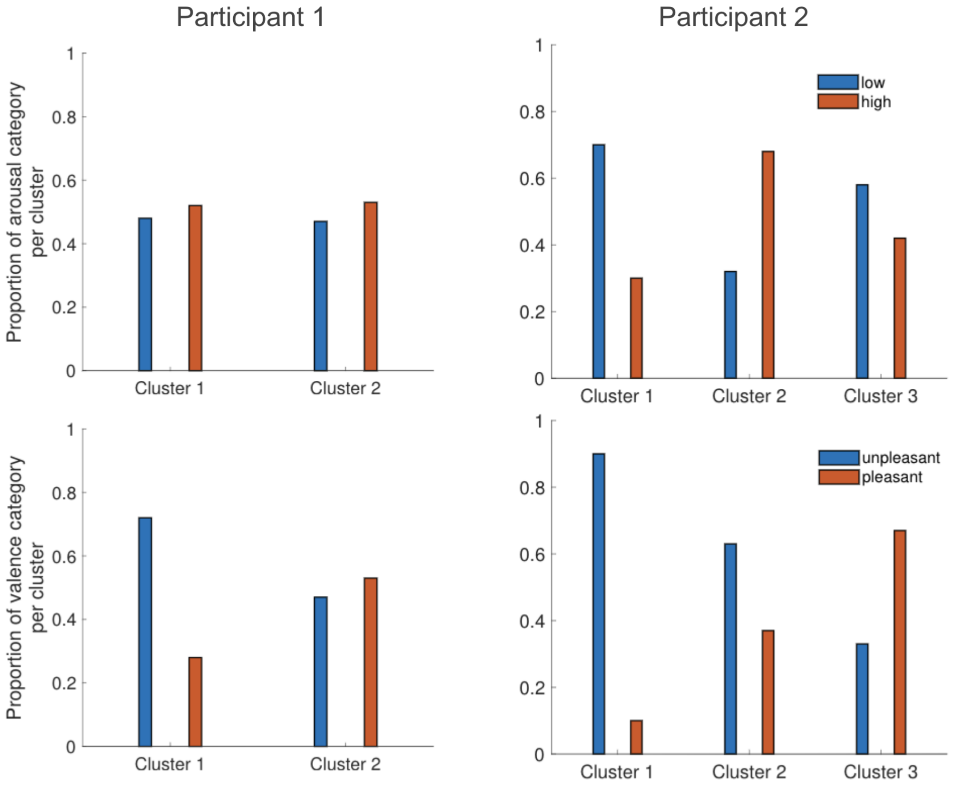

Supplementary Figure 2: **[BOLD Data] Correspondence between clusters and valence/arousal features for BOLD data from the 9s scenario immersion.** Left/right columns show data for two example participants with two and three clusters, respectively. Top row shows results for arousal and bottom row for valence. Specifically in the top row the heights of the bars represent the proportion of low arousal (blue) and high arousal (red) trials in each cluster while in the bottom row the bars represent the proportion of unpleasant (blue) and pleasant (red) trials in each cluster. The total proportion in each cluster sums to 1.

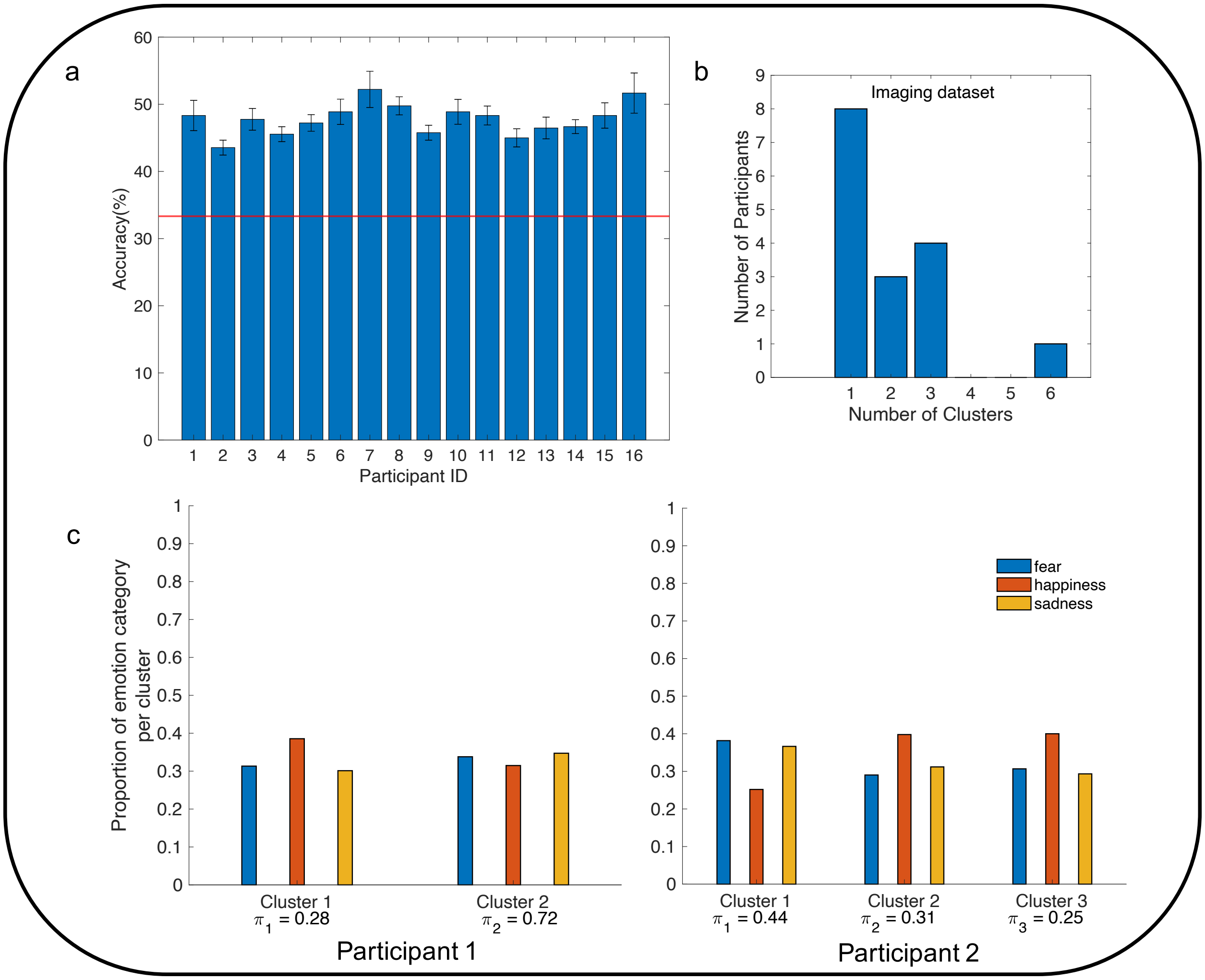

Supplementary Figure 3: **[BOLD Data] Results from supervised and unsupervised analyses of BOLD data from the 3s post-stimulus interval.** a) Supervised clustering: Mean +/- SEM classification accuracy from within-subject CNN supervised classification. The red line represents chance level accuracy (33.3%). b)-c) Unsupervised clustering: b) Histogram of number of participants fit by 1 through 6 GMM clusters. Specifically, eight participants had one discovered cluster, three participants had two clusters, four had three clusters, and one had six clusters. c) Correspondence between discovered clusters and emotion category labels for two example participants (corresponding to Participant IDs 1 and 2 in part a) with two (left) and three (right) discovered clusters respectively. Bars represent the proportion of the trials from each emotion category found within each cluster. Blue bars represent trials labeled as fear, orange bars happiness, and yellow sadness. The total proportion of categories in each cluster sums to 1. The mixing proportion π_k reported below each cluster is the probability that an observation comes from that cluster, which is representative of the size of the cluster.

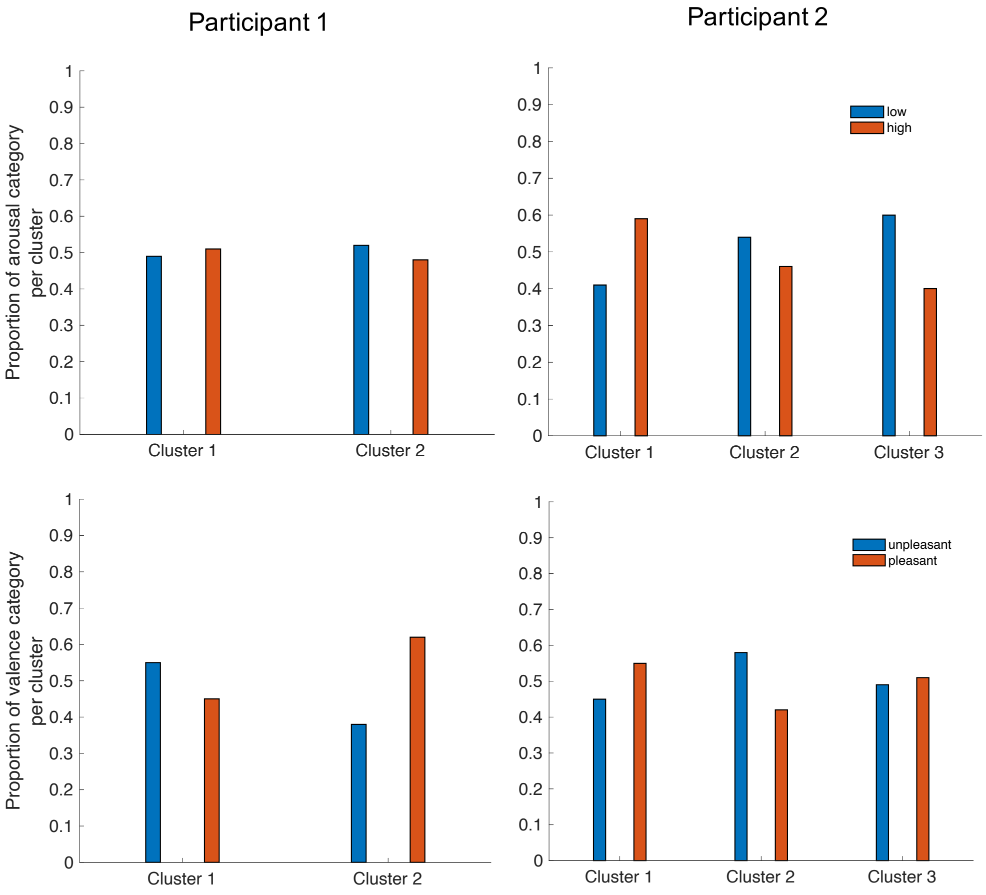

Supplementary Figure 4: **[BOLD Data] Correspondence between clusters and valence/arousal features for BOLD data from the 3s post-stimulus interval.** Left/right columns show data for two example participants corresponding to Participant IDs 1 and 2 with two and three clusters, respectively. Top row shows results for arousal and bottom row for valence. Specifically in the top row the heights of the bars represent the proportion of low arousal (blue) and high arousal (red) trials in each cluster while in the bottom row the bars represent the proportion of unpleasant (blue) and pleasant (red) trials in each cluster. The total proportion in each cluster sums to 1.

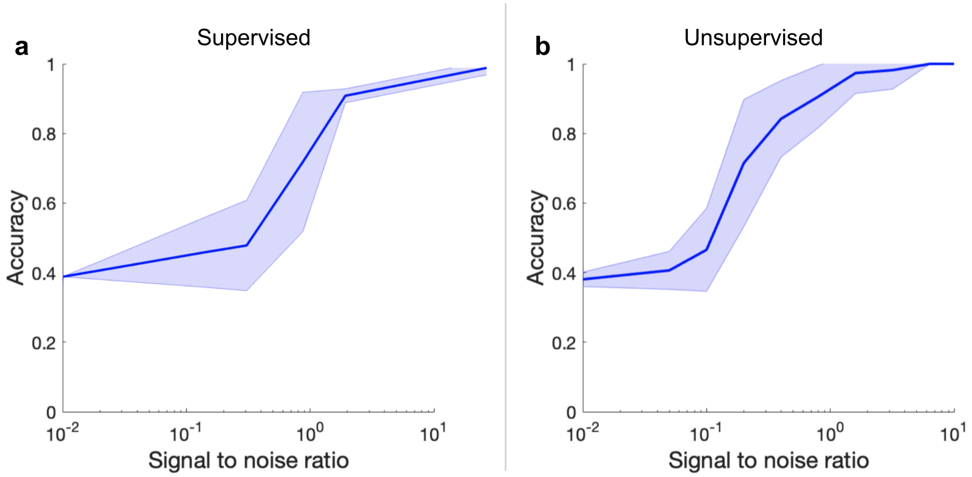

Supplementary Figure 5: **[Synthetic BOLD Data] Evaluating modeling performance using synthetic data.** Accuracy for supervised classification (left, (a)) and unsupervised clustering (right, (b)) for increasing SNR for the synthetic dataset generated based on the assumption that unique patterns of brain activity exists for different emotion categories. Unsupervised clustering accuracy represents the correspondence of the unsupervised clusters with the category labels, i.e., each category is associated to the most probable cluster according to the GMM. Shaded bands represent standard deviation.

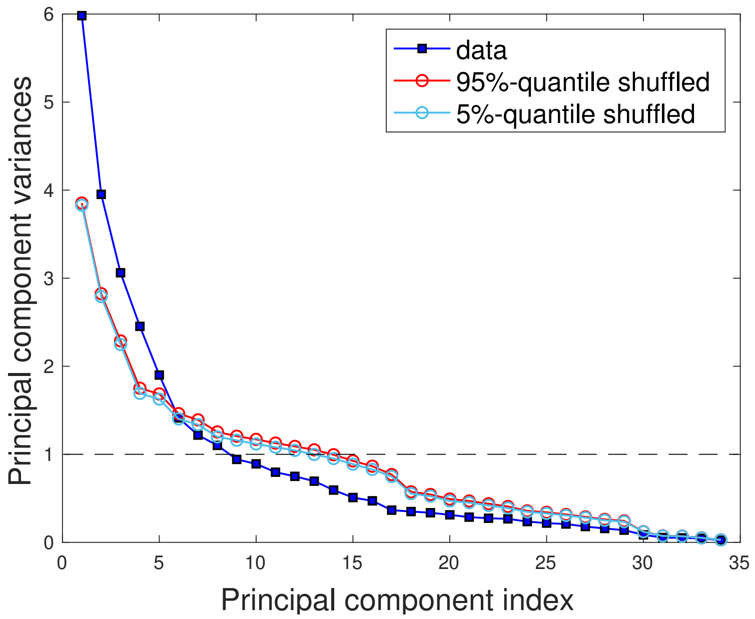

Supplementary Figure 6: **[Self-Report] Results from a principal component analysis of self-report data**. The plot depicts principle component variance (i.e. eigenvalues of the covariance matrix accounted for by each successive value) as a function of principle component index. Actual values are shown in blue squares; the line is drawn for better visualization. Different methods indicate different numbers of components to retain. The dashed line indicates a the retention criteria of eigenvalues greater than one, which indicates eight components. Alternately, a parallel analysis [5] to determine the number of components suggested five components, indicated by the 95% and 5% quantile of the distribution of shuffled data.

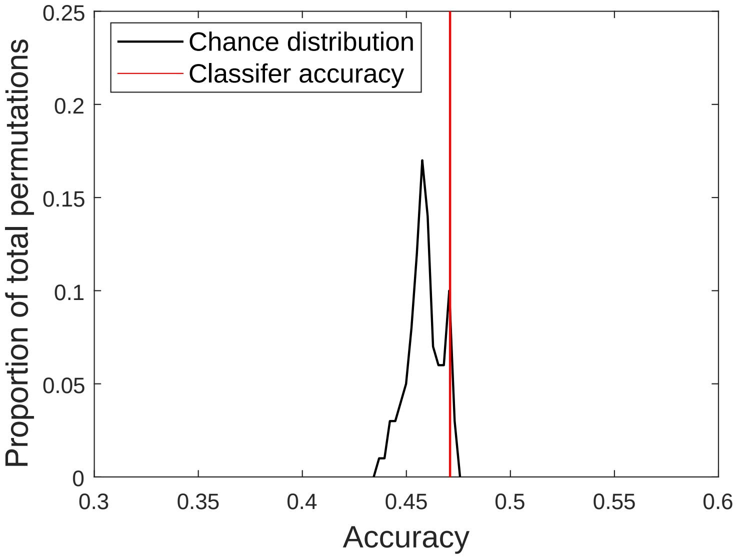

Supplementary Figure 7: **[ANS Data] Distribution of chance level accuracy obtained by the permutation test.** We ran 1000 permutations of classification on shuffled training labels and averaged classification accuracies across all participants for every permutation. The average classifier accuracy across subjects was 47.1%, which fell within the 5% tail of the chance distribution, indicating statistical significance.

References

[1] L. F. Barrett, Z. Khan, J. Dy, and D. Brooks. Nature of emotion categories: Comment on cowen and keltner. *Trends in Cognitive Sciences*, 22(2):97–99, 2018.

[2] A. S. Cowen and D. Keltner. Self-report captures 27 distinct categories of emotion bridged by continuous gradients. *Proceedings of the National Academy of Sciences*, 114(38):E7900–E7909, 2017.

[3] P. Good. *Permutation tests: a practical guide to resampling methods for testing hypotheses.* Springer Science & Business Media, 2013.

[4] T. R. L. Griffiths and M. Steyvers. Finding scientific topics. *Proceedings of the National Academy of Sciences*, 101:5228–5235, 2004.

[5] J. L. Horn. A rationale and test for the number of factors in factor analysis. *Psychometrika*, 30(2): 179–185, 1965.

[6] D. P. Kingma and J. Ba. Adam: A method for stochastic optimization. *arXiv preprint arXiv:1412.6980*, 2014.

[7] J. R. Manning, R. Ranganath, K. A. Norman, and D. M. Blei. Topographic factor analysis: A bayesian model for inferring brain networks from neural data. *PLoS One*, 9(5):e94914, 2014.

[8] N. Srivastava, G. Hinton, A. Krizhevsky, I. Sutskever, and R. Salakhutdinov. Dropout: A simple way to prevent neural networks from overfitting. *Journal of Machine Learning Research*, 15(56):1929–1958, 2014.
